# Propyrisulfuron plus cyhalofop butyl as one-shot herbicides provide high weed control efficiency and net economic performance in mechanically transplanted rice

**DOI:** 10.3389/fpls.2023.1281931

**Published:** 2023-10-18

**Authors:** Zichang Zhang, Hongchun Wang, Tao Gu, Jingjing Cao, Yuanlai Lou, Gui Li

**Affiliations:** Institute of Plant Protection, Jiangsu Academy of Agricultural Sciences, Nanjing, Jiangsu, China

**Keywords:** propyrisulfuron, cyhalofop butyl, mechanically transplanted rice, weed control, economic performance

## Abstract

Propyrisulfuron is a novel pyrimidinylsulfonylurea herbicide with good activity for controlling annual weed in rice fields. To evaluate the economic performance of propyrisulfuron, a field study was conducted in 2021 and 2022 on a farm of the Jiangsu Academy of Agricultural Sciences, China. Eight different herbicide treatments were employed, including CB (cyhalofop butyl), Py (propyrisulfuron), CBPy (cyhalofop butyl plus propyrisulfuron), PrBe 3, PrBe 10, and PrBe 3+PrBe 10 (pretilachlor plus bensulfuron applied at different times [at 3 (PrBe 3) and 10 (PrBe 10) d] or sequentially, respectively), 2PrBe+PeCBBz (pretilachlor plus bensulfuron [applied sequentially] followed by penoxsulam plus cyhalofop butyl plus bentazone), 2PrBe+MeCBBz (pretilachlor plus bensulfuron [applied sequentially] followed by metamifop plus cyhalofop butyl plus bentazone), along with weed-free and nontreated weedy check treatments. Herbicide treatments did not cause visual phytotoxicity to rice, and bending and leaf rolling were not observed. Only the two propyrisulfuron treatments had temporary negative effects on rice height, but rice recovered quickly. Compared with the weed-free treatment, CBPy did not affect rice tiller number or dry matter accumulation. Compared with the nontreated weedy check, herbicide treatments reduced total weed density by 29.4% to 99.1% and dry biomass by 32.2% to 98.7%. The CBPy treatment provided the best weed control, reducing weed density and biomass by 96.7% and 95.9% in 2021 and 97.4% and 95.6% in 2022, respectively. Rice grain yield was not significantly different between CBPy and the weed-free treatment in either year. Economic analysis showed that CBPy provided the highest net profit, followed by that in 2PrBe+PeCBBz and 2PrBe+MeCBBz, with the lowest net profit in the nontreated weedy check. Thus, CBPy provides good weed control and could be promoted in mechanically transplanted rice fields in China.

## 1 Introduction

Rice (*Oryza sativa* L.) is a major staple food for more than half the global population (Kumar and Ladha, 2011). Approximately 90% of the global rice supply is grown and consumed in Asia (Chauhan and Abugho, 2013). Rice is largely grown by manual transplanting, which is labor-intensive, dull and tedious, and is also very expensive (Shivashenkaramurthy et al., 2020). In recent years, urban expansion and migration of rural labor has resulted in labor scarcity and an increase in wages, especially in China (Raj and Elizabeth, 2017; Yuan et al., 2018).

To solve the labor shortage and reduce costs, rice establishment has shifted from manual transplanting to mechanized transplanting (Huang and Zou, 2018). Mechanical transplanting of rice is considered the most promising option, because it saves labor, ensures timely transplanting, and attains optimum plant density that contributes to productivity (Sathish et al., 2017). However, the occurrence of a large number of weeds in machine transplanted fields has become an important factor limiting the large-scale promotion of this planting method. Several reasons are responsible for the proliferation of weeds in machine-transplanted rice fields. First, the rice seedlings used for machine transplanting are relatively small, taking approximately 18 days from sowing to transplanting. Additionally, they often undergo alternating wet and dry irrigation management, which creates favorable conditions for weed emergence (Fang, 2001). Second, because mechanically transplanted rice seedlings are fragile, and may be injured in the process of mechanized transplanting, which delayed the application of herbicides. Furthermore, weed germination also benefits from the large row spacing (25–35 cm) in mechanized transplanting (Kolb et al., 2012; Dass et al., 2017), which increases the challenge of implementing effective weed control. Therefore, proper weed management is one of the most important prerequisites to ensure high crop yields in mechanized transplanted rice systems.

Chemical management is the most popular method of weed control in rice, because it is inexpensive, reliable, and labor- and time-saving (Rao et al., 2007; Gaines et al., 2021). Both pre-emergence (PRE) and post-emergence (POST) herbicides are commonly used to achieve optimal weed control in mechanically transplanted rice fields. Sole or sequential application of PRE pretilachlor plus bensulfuron, oxadiargyl, and butachlor provides effective weed control (Chauhan and Opeña, 2012; Teja et al., 2015; Bajwa et al., 2019). However, some problems are associated with use of PRE herbicides, such as a limited time window for application, short-duration weed control, and resistance (Singh et al., 2006; Deng et al., 2021). When weed control efficiency is not satisfactory at the early stage of rice, POST herbicides may be a better option to manage weeds (Singh et al., 2006; Mahajan and Chauhan, 2013).

Approximately 60% of weeds emerge during 7–30 days after transplanting and greatly compete with rice (Saha and Rao, 2010). In China, the most popular PRE herbicide option for controlling weeds in mechanically transplanted rice is a combination of pretilachlor plus bensulfuron, commonly applied twice (3 d before mechanical transplanting and then 10 d after transplanting). Because PRE pretilachlor plus bensulfuron do not offer long-lasting effectiveness, POST herbicides, such as penoxsulam, metamifop, cyhalofop butyl, and bentazone, are also applied at the rice tilling stage. Sequential applications of PRE and POST herbicides provide very effective weed control in mechanized transplanting of rice (Qiu et al., 2016; Zhu et al., 2020). However, it is also important to consider the economic cost of weed control and whether there are other alternative low-cost and high-efficiency control PRE and POST herbicides that can reduce application times and evolution of herbicide resistance. Unfortunately, such information is not available for the herbicides used in mechanized transplanting of rice in China.

Propyrisulfuron is a pyrimidinylsulfonylurea herbicide developed in 2008 for weed control in rice, and it has high activity not only against broadleaf weeds and sedges but also against grasses (Ikeda et al., 2011). Propyrisulfuron has a fused heterocyclic moiety bonded to a sulfonyl group, a property that provides high activity against sulfonylurea-resistant weeds (Tanaka et al., 2006; Ikeda et al., 2011). However, propyrisulfuron is not widely used in rice fields in China. To evaluate the performance of propyrisulfuron in China, a field experiment was conducted that compared safety to rice, weed control efficacy, yield, and economics of propyrisulfuron with those of herbicide weed management practices currently used by farmers in mechanically transplanted rice.

## 2 Materials and methods

### 2.1 Experimental site description

**Figure 1:**
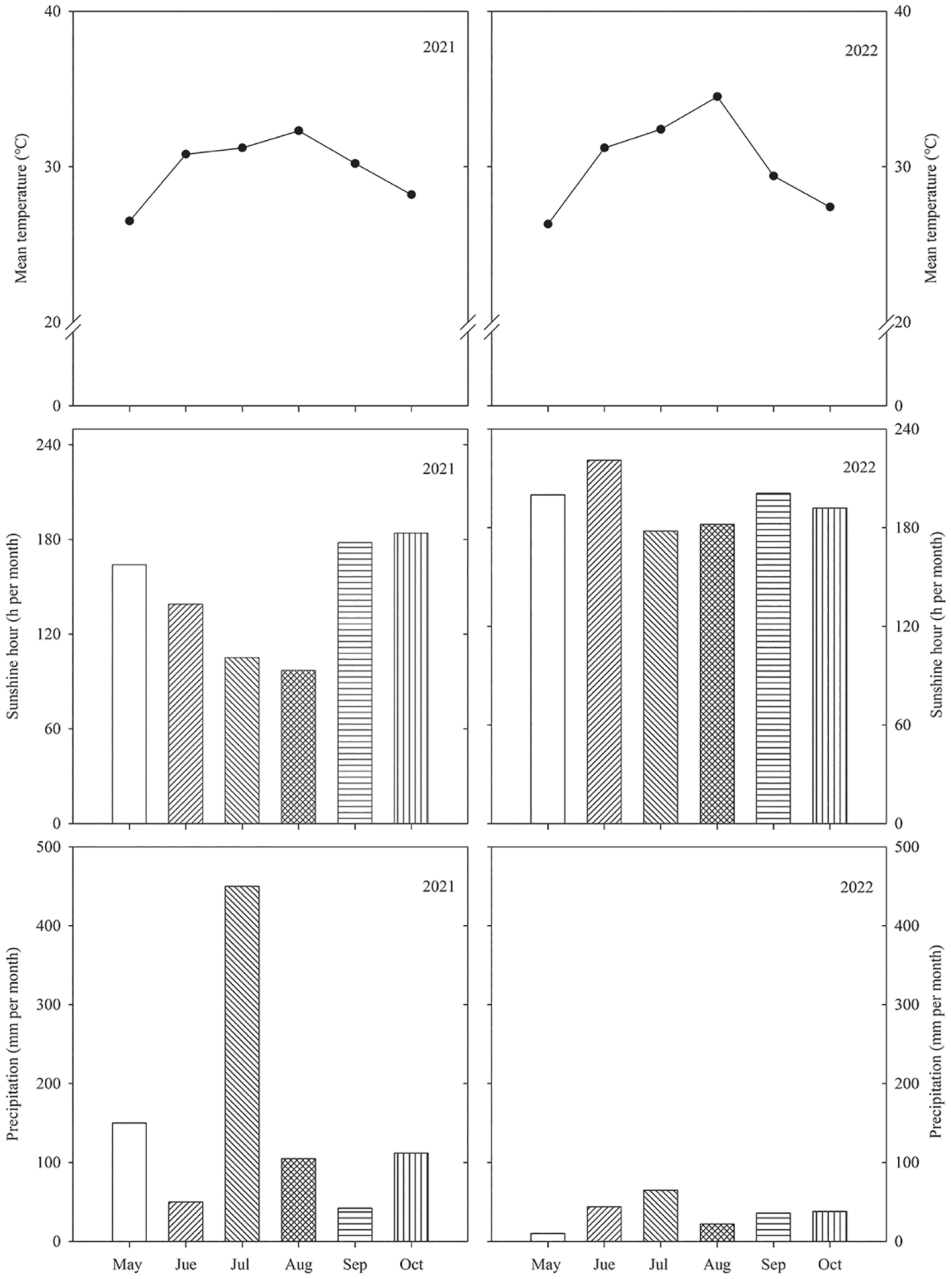
Precipitation, sunshine hours, and mean temperature during the growing season of rice across the 2 years (2021–2022) in Nanjing, Southeast China. Precipitation, sunshine hours are monthly totals. Temperatures are the monthly averages.

To evaluate herbicides, an experiment was conducted on a farm at the Jiangsu Academy of Agricultural Sciences, Jiangsu Province, China (32°18′N, 118°52′E), during the rice growing season (May–October) in 2021 and 2022. The soil was an Entisol, Typic fluvaquent (Baldwin et al., 1938) and a sandy loam with 21.2 g kg ^−1^ organic matter, 1.18 g kg^−1^ total nitrogen (N), 32.4 mg kg^−1^ Olsen-phosphorus, and 67.2 mg kg ^−1^ exchangeable potassium (K). The average air temperature, precipitation, and sunshine hours during the rice-growing season across the two years were recorded at a weather station close to the experimental site, as listed in **Figure 1**.

The high-yielding rice cultivar Nanjing 9108 (a *japonica* cultivar), which is currently used in local production, was grown in the paddy field in 2021 and in 2022. In both years, seedlings were raised in a seedbed with sowing date on 15–16 May and mechanically transplanted on 3–5 June at a hill spacing of 0.25 m × 0.14 m with three seedlings per hill. Heading date (50% of plants) was on 25–28 August, and plants were harvested on 15–18 October. Total N application rate was 270 kg ha^−1^, and N was applied as urea 1 d before transplanting and at early tillering (7 d after transplanting [DAT]) and panicle initiation (40 DAT) stages in proportions of 50%, 10%, and 40%, respectively. Phosphorus (40 kg ha^−1^ as single superphosphate) and K (40 kg ha^−1^ as KCl) were applied as basal fertilizer the day before mechanical transplanting. The experiment followed a complete randomized block design, with four replicates and a plot size of 4 m × 6 m. Plots were separated by a round 0.5 m wide alley using plastic film inserted into soil to a depth of 50 cm.

### 2.2 Treatments

The experiment comprised ten treatments listed in **Table 1** and follows: (1) cyhalofop butyl (CB, Jiangsu Suke Agricultural Chemical Co., Ltd, Nanjing, China), which is an aromatic oxyphenoxypropionic acid herbicide widely used in rice fields for grass weed control, especially for *Leptochloa chinensis*; (2) propyrisulfuron (Py, Sumitomo Chemical Co., Ltd, Chuo-ku, Japan), which is a novel sulfonylurea herbicide that is widely used in controlling annual or perennial weeds and grasses in paddy fields because of its high safety, high efficiency, and broad-spectrum yet highly selective effects; (3) cyhalofop butyl+propyrisulfuron (CBPy); (4) pretilachlor+bensulfuron (PrBe 3, herbicide was mixed with soil and broadcasted manually at 3 d before transplanting; Jiangsu Kuaida Agrochemical Co., Ltd, Nantong, China), which is a mixture of sulfonylurea and amide herbicides that interferes with amino acid and protein synthesis in annual weeds; (5) pretilachlor+bensulfuron (PrBe 10, applied at 10 DAT); (6) pretilachlor+bensulfuron followed by (fb) pretilachlor+bensulfuron (PrBe 3+PrBe 10); (7) pretilachlor+bensulfuron fb pretilachlor+bensulfuron fb penoxsulam+cyhalofop butyl+bentazone (2PrBe+PeCBBz), with penoxsulam (Dow AgroSciences, Shanghai, China) an acetolactate synthase inhibitor that is a highly efficient, broad-spectrum herbicide used in rice fields worldwide and bentazone (Jiangsu Institute of Ecomones Co., Ltd, Jintan Jiangsu, China) a POST herbicide used to treat broad-leaf weeds in a variety of crops including rice; (8) pretilachlor+bensulfuron fb pretilachlor+bensulfuron fb metamifop+cyhalofop butyl+bentazone (2PrBe+MeCBBz), with metamifop (FMC Suzhou Crop Care Co., Ltd, Suzhou, China) a POST aryloxyphenoxypropionic acid herbicide used to control a wide range of annual and perennial grass weeds in rice; (9) weed-free (W0), which was manually weeded; (10) nontreated weedy check (W+). The POST herbicides (**Table 1**) were sprayed using a CO_2_-pressurized knapsack sprayer equipped with four “teejet 8002” (TeeJet Technologies (Ningbo) Co., Ltd, Ningbo, Zhejiang, China) flat-fan nozzles to deliver water at 450 L ha^−1^. Spray pressure, spraying height, boom width, and nozzle spacing were 2.0 bar, 0.5 m, 2.0 m, and 0.5 m, respectively.

**Table 1:** Timing and rate of herbicide treatments in mechanically transplanted rice.

Treatment		Treatment application
Herbicide	abbreviation	
Cyhalofop butyl	CB	Cyhalofop butyl at 300 g a.i. ha^−1^ as POST at 12 DAT. Herbicide was mixed with clean water at 450 L ha^−1^ and sprayed using a CO_2_-pressurized knapsack.
Propyrisulfuron	Py	Propyrisulfuron at 85.5 g a.i. ha^−1^ as POST at 12 DAT. Application method was the same as in CB.
Cyhalofop butyl + propyrisulfuron	CBPy	Cyhalofop butyl at 300 g a.i. ha^−1^ plus Py at 85.5 g a.i. ha^−1^ as POST at 12 DAT. Application method was the same as in CB.
Pretilachlor + bensulfuron	PrBe3	Pretilachlor + bensulfuron at 420 g a.i. ha^−1^ as PRE at 3 DBT. Herbicide was mixed with soil and broadcasted manually.
Pretilachlor + bensulfuron	PrBe10	Pretilachlor + bensulfuron at 420 g a.i. ha^−1^ as PRE at 10 DAT. Application method was the same as in PrBe3.
Pretilachlor + bensulfuron fb pretilachlor + bensulfuron	PrBe3+PrBe10	Pretilachlor + bensulfuron at 420 g a.i. ha^−1^ as PRE at 3 DAT and 10 DAT. Application method was the same as in PrBe3.
Pretilachlor + bensulfuron fb pretilachlor + bensulfuron fb penoxsulam + cyhalofop butyl + bentazone	2PrBe+PeCBBz	Pretilachlor + bensulfuron at 420 g a.i. ha^−1^ as PRE at 3 DAT and 10 DAT fb penoxsulam at 37.5 a.i. ha^−1^ plus CB at 300 g a.i. plus bentazone g a.i. as POST at 30 DAT. Application method of PrBe was the same as in PrBe3 and the other herbicides (premix) application method was the same as in CB.
Pretilachlor + bensulfuron fb pretilachlor + bensulfuron fb metamifop + cyhalofop butyl + bentazone	2PrBe+MeCBBz	Pretilachlor + bensulfuron at 420 g a.i. ha^−1^ as PRE at 3 DAT and 10 DAT fb metamifop at 300 a.i. ha^−1^ plus CB at 300 g a.i. plus BZg a.i. as POST at 30 DAT. Application method of PrBe was the same as in PrBe3 and the other herbicides (premix) application method was the same as in CB.
Weed-free	W0	Plots kept weed-free by frequent manual weeding.
Nontreated weedy check	W+	No weeding.

a.i., active ingredient; fb, followed by; DAT, days after transplanting; DBT, days before transplanting.

### 2.3 Treatment evaluation

#### 2.3.1 Crop safety

Symptoms of rice phytotoxicity were assessed at 3, 5, 7, 10, and 15 DAT using a visual rating scale (Rahman et al., 2011): 1 = highly resistant (green shoot and leaves); 2 = resistant (green shoot and light leaves); 3 = partly resistant (green shoot and pale yellow leaves); 4 = susceptible (almost dead); and 5 = highly susceptible (dead). Plant height was also measured from the soil surface to the tip of the uppermost leaf every 10 d after mechanical transplanting. Rice tillers were counted in each plot in a 1.0 m^2^ area of rice seedlings every 10 d until 80 DAT. In addition, aboveground rice biomass was recorded in each plot in a 0.25 m^2^ area at 0, 20, and 40 DAT.

#### 2.3.2 Weed control efficacy

Weed density levels were recorded in four randomly located quadrats (0.5 m × 0.5 m) in each plot 15, 30, and 60 DAT. Aboveground parts of all dominant weeds were collected at 60 DAT and then oven-dried at 70°C for 72 h to determine dry weight. Weed control efficacy (WCE) was calculated by the following formulas:


$$\begin{array}{l} \text{Efficacy of weed density control }(\%)=(\text{weed number in untreated plot}-\text{weed number in herbicide}-\text{treated plot})/\\ \text{weed number in untreated plot}\times 100; \end{array}$$

$$\begin{array}{l} \text{Efficacy of weed> }\mathrm{aboveground}\text{ biomass control }(\%)=(\text{weed }\mathrm{aboveground}\text{ biomass in untreated plot}-\\ \text{weed }\mathrm{aboveground}\text{ biomass in herbicide}-\text{treated plot})/\text{weed }\mathrm{aboveground}\text{ biomass in untreated plot}\times 100 \end{array}$$

#### 2.3.3 Rice yield and net economic returns

Grain yields were recorded from a 5-m^2^ site (except border plants and weed sampling areas) in each plot then were adjusted to a moisture content of 0.14 g of H_2_O g^−1^ fresh weight before statistical analysis.

Net returns for each treatment were calculated over variable costs of production (**Table 2**). Inputs such as pesticides/fertilizers/seeds for each year were all purchased from the same source. Irrigation was provided with electric motor pump sets with fixed electricity charges. The cost of human labor for tillage, seeding, irrigation, fertilizer and pesticide application, weeding, and harvesting of crops was based on actual cost to farmers and was estimated considering total acreage and person-hours. All costs were summed to calculate total variable cost of production. All costs were in Chinese Yuan and dollars, respectively, and were averaged over the two years.

**Table 2:** Variable costs for farm operations, herbicides, and other costs in mechanically transplanted rice.

Operations/inputs	Cost	
	(Yuan ha^−1^)	($ ha^–1^)
Tillage	1500	209.25
Seed cost	187.5	26.16
Mechanical transplanting	120	16.74
Pre-emergent herbicides		
Pretilachlor+bensulfuron	150	20.93
Post-emergent herbicides		
Cyhalofop butyl	90	12.56
Propyrisulfuron	325	45.34
Penoxsulam	300	41.85
Bentazone	240	33.48
Metamifop	270	37.67
Other costs		
Hand weeding for weed-free check	6750	941.63
Fertilizers	5550	774.23
Fungicides	450	62.78
Insecticides	300	41.85
Electricity charges	225	31.39
Permanent labor charges	1155	161.12
Harvesting cost	750	104.63
Sale price of rice per tonne	3200	446.4

The conversion rate of Yuan to dollars was 0.1395 (1 dollar = 7.1677 Yuan).

### 2.4 Statistical analyses

Analysis of variance was performed using a SAS/STAT statistical analysis package (v6.12, SAS Institute, Cary, NC, USA). Data from each sampling date were analyzed separately. Means were tested by the least significant difference at *P* = 0.05.

## 3 Results

### 3.1 Weed species

Weeds in the experimental field were a naturally occurring mixed population. In W+ (nontreated weedy check), there were 11 weed species dominated by grass, broadleaf, and sedge weeds. The dominant weeds were two grass species: *Echinochloa* spp. and *Leptochloa chinensis* (L.) Nees; two broadleaf species: *Monochoria vaginalis* (Burm.f.) C. Presl and *Ammannia multiflora* Roxb; and one sedge species: *Cyperus difformis* L.

### 3.2 Crop injury

**Figure 2:**
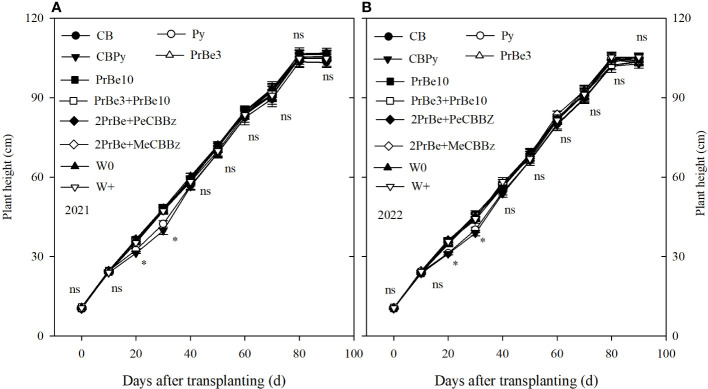
Height of mechanically transplanted Nanjing 9108 rice plants after application of different herbicide treatments in **(A)** 2021 and **(B)** 2022. CB, Cyhalofop butyl; Py, Propyrisulfuron; CBPy, Cyhalofop butyl + propyrisulfuron; PrBe3, Pretilachlor + bensulfuron applied at 3 d before transplanting; PrBe10, applied at 10 d after transplanting; PrBe3+PrBe10, two applications of pretilachor+bensulfuron (at 3 [PrBe3] and 10 [PrBe10] d]; 2PrBe+PeCBBz, two applications of pretilachor+bensulfuron (at 3 [PrBe3] and 10 [PrBe10] d) plus penosulam plus CB plus bentazone; 2PrBe+MeCBBz, two applications of pretilachor+bensulfuron (at 3 [PrBe3] and 10 [PrBe10] d) plus metamifop plus CB plus BZ; W0, plots kept weed-free by frequent manual weeding; W+, nontreated weedy check. Error bars denote ± standard errors of the mean (n = 4). Those marked with asterisk are significantly different at the 0.05 probability; those with ns are not significantly different within the same period of measurement.

All herbicides had a visual phytotoxicity rating of 1 (green shoot and leaves) and bending and leaf rolling were not observed (data not shown). The herbicide treatments CB, PrBe 3, PrBe 10, PrBe 3+PrBe 10, 2PrBe+PeCBBz, and 2PrBe+MeCBBz did not affect plant height (**Figure 2**), and Py and CBPy had only early negative effects. Although height was reduced by 11.3% to 13.6% in Py and by 14.1% to 17.60% in CBPy at 20 and 30 DAT, respectively, there were no differences in final plant height between the two herbicide treatments and W0 (weed-free treatment) in 2021. Results were similar in 2022. Therefore, although application of Py at 85.5 g a.i. ha^−1^ had a temporary negative effect on rice height, by 60 DAT, final rice height was not affected (**Figure 2**).

**Figure 3:**
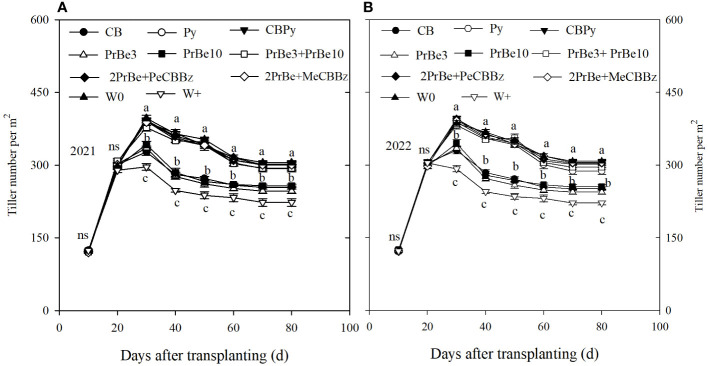
Tiller number of mechanically transplanted Nanjing 9108 rice plants after application of different herbicide treatments in **(A)** 2021 and **(B)** 2022. CB, Cyhalofop butyl; Py, Propyrisulfuron; CBPy, Cyhalofop butyl + propyrisulfuron; PrBe3, Pretilachlor + bensulfuron applied at 3 d before transplanting; PrBe10, applied at 10 d after transplanting; PrBe3+PrBe10, two applications of pretilachor+bensulfuron (at 3 [PrBe3] and 10 [PrBe10] d]; 2PrBe+PeCBBz, two applications of pretilachor+bensulfuron (at 3 [PrBe3] and 10 [PrBe10] d) plus penosulam plus CB plus bentazone; 2PrBe+MeCBBz, two applications of pretilachor+bensulfuron (at 3 [PrBe3] and 10 [PrBe10] d) plus metamifop plus CB plus BZ; W0, plots kept weed-free by frequent manual weeding; W+, nontreated weedy check. Error bars denote ± standard errors of the mean (n = 4). Different letters indicate a significant difference at the 0.05 probability level.

In the two years, tillering number was not significantly different among treatments before 20 DAT, but with continued rice growth, significant differences were observed among treatments (**Figure 3**). Tiller numbers in herbicide treatments were higher than those in W+. Compared with W0, tiller number in CB, PrBe3, and PrBe10 decreased significantly, whereas number in other herbicide treatments, including CBPy, was not significantly different. Thus, CBPy treatment had no significant effect on rice tillering number (**Figure 3**).

**Figure 4:**
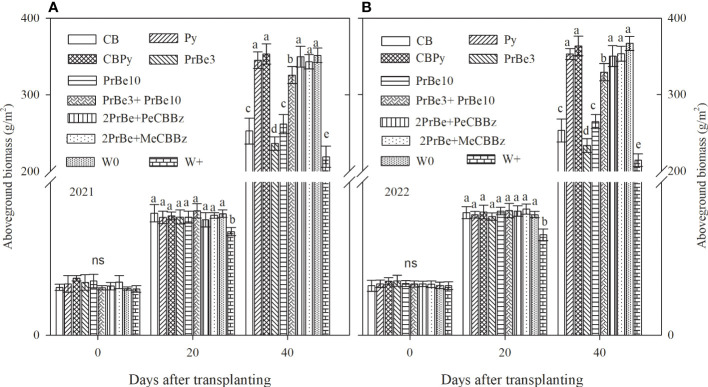
Aboveground biomass of mechanically transplanted Nanjing 9108 rice plants after application of different herbicide treatments in **(A)** 2021 and **(B)** 2022. CB, Cyhalofop butyl; Py, Propyrisulfuron; CBPy, Cyhalofop butyl + propyrisulfuron; PrBe3, Pretilachlor + bensulfuron applied at 3 d before transplanting; PrBe10, applied at 10 d after transplanting; PrBe3+PrBe10, two applications of pretilachor+bensulfuron (at 3 [PrBe3] and 10 [PrBe10] d]; 2PrBe+PeCBBz, two applications of pretilachor+bensulfuron (at 3 [PrBe3] and 10 [PrBe10] d) plus penosulam plus CB plus bentazone; 2PrBe+MeCBBz, two applications of pretilachor+bensulfuron (at 3 [PrBe3] and 10 [PrBe10] d) plus metamifop plus CB plus BZ; W0, plots kept weed-free by frequent manual weeding; W+, nontreated weedy check. Error bars denote ± standard errors of the mean (n = 4). Different letters indicate a significant difference at the 0.05 probability level, and those with ns are not significantly different within the same period of measurement.

Compared with W0, rice aboveground biomass decreased in W+ but was not significantly different in the other treatments at 20 DAT (**Figure 4**). However, at 40 DAT, aboveground biomass was significantly higher in Py, CBPy, PrBe 3+PrBe 10, 2PrBe+PeCBBz, 2PrBe+MeCBBz, and W0 than in CB, PrBe 3, and PrBe10. It is worth noting that the CBPy treatment had no significant effect on rice aboveground biomass compared to the W0 treatment (**Figure 4**).

### 3.3 Weed control efficacy

At 15 DAT in both 2021 and 2022, compared with W+, densities of the dominant weed species *Echinochloa* spp., *L. chinensis*, *M. vaginalis*, *A. multiflora*, and *C. difformis* were not significantly affected in CB, Py, and CBPy with post-emergence spraying (**Table 3**). it was only 3 d after herbicide application, and effects had not appeared. However, compared with W+, densities of dominant weeds decreased significantly in PrBe 3, PrBe 10, PrBe 3+PrBe 10, 2PrBe+PeCBBz, and 2PrBe+MeCBBz, especially in PrBe 3+PrBe 10, 2PrBe+PeCBBz, and 2PrBe+MeCBBz with 100% weed control efficiency. Besides, the total weed control efficiency of the three treatments were also 100%. In PrBe 3 and PrBe 10, efficacy of weed density control was 91.2% and 98.5% in 2021 and 93.1% and 98.3% in 2022, respectively (**Table 3**).

**Table 3:** Effects of herbicide treatments on densities (m^−2^) of dominant weeds at 15 d after mechanical transplanting of rice in 2021 and 2022.

Year	Treatment	*Echinochloa* spp.	*Leptochloa chinensis*	*Monochoria vaginalis*	*Ammannia multiflora*	*Cyperus difformis*	Total weed density	Efficacy of weed density control (%)
2021	CB	66.2a	30.4a	10.8a	14.2a	22.4a	144.0a	3.9
	Py	65.4a	34.8a	8.4a	15.2a	16.8a	140.6a	6.2
	CBPy	69.2a	32.2a	9.2a	16.4a	19.6a	141a	5.9
	PrBe3	4.8b	2.8b	2.0b	0.8b	1.6b	12b	91.2
	PrBe10	1.4b	0.8b	0.0c	0.0b	0.0b	2.2b	98.5
	PrBe3+PrBe10	0.0b	0.0c	0.0c	0.0b	0.0b	0.0c	100
	2PrBe+PeCBBz	0.0c	0.0c	0.0c	0.0b	0.0b	0.0c	100
	2PrBe+MeCBBz	0.0c	0.0c	0.0c	0.0b	0.0b	0.0c	100
	W+	68.4a	32.2a	12.6a	16.3a	20.4a	149.9a	
2022	CB	69.2a	28.2a	12.1a	11.6a	26.7a	147.8a	2.1
	Py	67.9a	31.3a	11.4a	13.8a	22.9a	147.3a	2.4
	CBPy	65.3a	33.7a	11.9a	11.4a	23.4a	145.7a	3.5
	PrBe3	5.2b	2.4b	1.3b	0.3b	1.2b	10.4b	93.1
	PrBe10	1.2b	1.4b	0.0b	0.0b	0.0b	2.6c	98.3
	PrBe3+PrBe10	0.0b	0.0b	0.0b	0.0b	0.0b	0.0c	100
	2PrBe+PeCBBz	0.0b	0.0b	0.0b	0.0b	0.0b	0.0c	100
	2PrBe+MeCBBz	0.0b	0.0b	0.0b	0.0b	0.0b	0.0c	100
	W+	70.3a	30.6a	13.6a	14.6a	21.8a	150.9a	

CB, Cyhalofop butyl; Py, Propyrisulfuron; CBPy, Cyhalofop butyl + propyrisulfuron; PrBe3, Pretilachlor + bensulfuron applied at 3 d before transplanting; PrBe10, applied at 10 d after transplanting; PrBe3+PrBe10, two applications of pretilachor+bensulfuron (at 3 [PrBe3] and 10 [PrBe10] d]; 2PrBe+PeCBBz, two applications of pretilachor+bensulfuron (at 3 [PrBe3] and 10 [PrBe10] d) plus penosulam plus CB plus bentazone; 2PrBe+MeCBBz, two applications of pretilachor+bensulfuron (at 3 [PrBe3] and 10 [PrBe10] d) plus metamifop plus CB plus BZ; W+, nontreated weedy check. Different letters within the same column and year indicate significant differences at P = 0.05.

At 30 DAT, herbicide treatments significantly reduced total weed density, although there were significant differences among treatments (**Table 4**). For average total weed density over two years, the lowest total weed density was in CBPy (0.6 in 2021 and 0.8 in 2022), followed by that in 2PrBe+MeCBBz (26.7 in 2021 and 28.0 in 2022), 2PrBe+PeCBBz (25.3 in 2021 and 30.1 in 2022), PrBe 3+PrBe 10 (26.2 in 2021 and 29.4 in 2022), Py (36.0 in 2021 and 39.0 in 2022), PrBe 10 (72.9 in 2021 and 79.7 in 2022), and PrBe 3 (135.1 in 2021 and 145.6 in 2022). The highest total weed density was in CB (106.6 in 2021 and 110.2 in 2022). Total weed control efficacy for different treatments reflected by the effects on total weed densities. Of the herbicide treatments, CBPy had the highest weed control of each weed species, providing almost complete control. Treatments PrBe 3+PrBe 10, 2PrBe+PeCBBz, and 2PrBe+MeCBBz also had relatively high efficacy of weed density control (>86%). In addition, we also found that there was no significant difference in *L. chinensis* density between Py and W+, indicating that Py was not effective against *L. chinensis*. The control efficacy of dominant weed species was similar in 2021 and 2022 (**Table 4**).

**Table 4:** Effects of herbicide treatments on densities (m^−2^) of dominant weeds at 30 d after mechanical transplanting of rice in 2021 and 2022.

Year	Treatment	*Echinochloa* spp.	*Leptochloa chinensis*	*Monochoria vaginalis*	*Ammannia multiflora*	*Cyperus difformis*	Total weed density	Efficacy of weed density control (%)
2021	CB	20.4d	5.8d	14.4a	35.6a	30.4a	106.6c	51.9d
	Py	1.2f	34.8a	0.0e	0.0e	0.0e	36.0e	83.8b
	CBPy	0.6f	0.0e	0.0e	0.0e	0.0e	0.6g	99.7a
	PrBe3	59.5b	22.0b	10.0b	22.1b	21.6b	135.1b	39.1e
	PrBe10	26.3c	14.3c	5.2c	14.5c	12.8c	72.9d	67.1c
	PrBe3+PrBe10	9.5e	1.2e	2.8d	5.9d	6.7d	26.2f	88.2b
	2PrBe+PeCBBz	10.5e	1.2e	1.9d	5.6d	6.0d	25.3f	88.6b
	2PrBe+MeCBBz	12.0e	1.5e	2.4d	3.6de	7.3d	26.7f	87.9b
	W+	104.8a	36.8a	13.8a	32.8a	33.6a	221.1a	
2022	CB	22.3c	7.2d	12.9a	33.1a	34.8a	110.2c	51.6d
	Py	1.4e	37.6a	0.0e	0.0e	0.0e	39.0e	82.9b
	CBPy	0.8e	0.0e	0.0e	0.0e	0.0e	0.8f	99.6a
	PrBe3	65.1b	24.7b	10.6b	23.4b	21.9b	145.6b	36.1e
	PrBe10	28.7c	12.3c	5.8c	18.0c	15.0c	79.7d	65.0c
	PrBe3+PrBe10	13.0d	1.8e	2.4d	3.4de	8.8d	29.4e	87.1b
	2PrBe+PeCBBz	11.3d	1.6e	2.1d	4.4d	10.8d	30.1e	86.8b
	2PrBe+MeCBBz	12.3d	0.5e	1.7de	3.1de	10.5d	28.0e	87.7b
	W+	108.1a	41.2a	13.2a	31.2a	34.3a	227.8a	

CB, Cyhalofop butyl; Py, Propyrisulfuron; CBPy, Cyhalofop butyl + propyrisulfuron; PrBe3, Pretilachlor + bensulfuron applied at 3 d before transplanting; PrBe10, applied at 10 d after transplanting; PrBe3+PrBe10, two applications of pretilachor+bensulfuron (at 3 [PrBe3] and 10 [PrBe10] d]; 2PrBe+PeCBBz, two applications of pretilachor+bensulfuron (at 3 [PrBe3] and 10 [PrBe10] d) plus penosulam plus CB plus bentazone; 2PrBe+MeCBBz, two applications of pretilachor+bensulfuron (at 3 [PrBe3] and 10 [PrBe10] d) plus metamifop plus CB plus BZ; W+, nontreated weedy check. Different letters within the same column and year indicate significant differences at P = 0.05.

At 60 DAT, compared with W+, total weed density control efficacy in 2021 was highest in 2PrBe+PeCBBz (98.9%), 2PrBe+MeCBBz (98.2%), and CBPy (96.7%), followed by that in Py (81.5%), PrBe 3+PrBe 10 (81.0%), and PrBe 10 (59.8%) (**Table 5**). The lowest control efficacy was in PrBe 3 (29.4%). The pattern of total weed dry weight control efficacy was similar among herbicide treatments, and corresponding values were 98.7% in 2PrBe+PeCBBz, 98.2% in 2PrBe+MeCBBz, 95.9% in CBPy, 85.8% in PrBe 3+PrBe 10, 83.1% in Py, 44.5% in CB, and 34.0% in PrBe3. Patterns for the five dominant weed species at 60 DAT were similar to those at 30 DAT. Results were generally consistent between years (**Table 5**).

**Table 5:** Effects of herbicide treatments on densities (m^−2^) and biomass (g m^−2^) of dominant weeds at 60 d after mechanical transplanting of rice in 2021 and 2022.

Year	Treatment	*Echinochloa* spp.		*Leptochloa chinensis*		*Monochoria vaginalis*		*Ammannia multiflora*		*Cyperus difformis*		Total weed density efficacy (%)	Total dry weight efficacy (%)
		Weed density	Dry weight	Weed density	Dry weight	Weed density	Dry weight	Weed density	Dry weight	Weed density	Dry weight		
2021	CB	44.3c	55.5c	6.4d	7.3c	15.9a	30.4a	35.5a	12.3a	37.8a	5.2a	43.7d	44.5d
	Py	2.4e	5.8f	35.2a	21.8a	1.4d	4.1c	2.8de	1.4d	2.3d	0.6d	81.5b	83.1b
	CBPy	1.9e	2.8f	0.6e	0.5d	1.2d	3.0cd	2.5de	1.6d	2.1d	0.4d	96.7a	95.9a
	PrBe3	80.6b	78.1b	27.8b	12.9b	13.7a	29.3a	26.8b	8.0b	26.6b	3.4b	29.4e	34.0e
	PrBe10	39.3c	44.6d	20.6c	8.7c	6.0c	20.8b	16.3c	5.1b	16.8c	2.0c	59.8c	59.3c
	PrBe3+PrBe10	24.8d	18.4e	6.8d	2.1d	2.3d	5.2c	6.8d	2.0d	4.5d	0.6d	81.0b	85.8b
	2PrBe+PeCBBz	0.8e	1.6f	0.8e	0.3d	0.3e	0.3d	1.0e	0.3d	0.8d	0.2d	98.9a	98.7a
	2PrBe+MeCBBz	1.25e	2.5f	0.5e	0.2d	0.3e	0.5d	1.3e	0.2d	1.3d	0.3d	98.2a	98.2a
	W+	116.8a	134.4a	39.4a	20.3a	14.2a	29.2a	37.8a	10.6a	40.4a	5.0a		
2022	CB	45.2c	69.9c	8.8d	8.1c	13.9a	37.4a	38.1a	14.4a	38.1a	5.6a	45.4e	42.2d
	Py	2.8e	6.6f	37.9a	27.2a	1.1dc	3.6cd	2.1e	1.9d	2.6d	0.4c	82.4c	83.1b
	CBPy	2.3e	5.5f	1.0e	0.8e	0.4c	2.0d	1.6e	1.7d	1.7d	0.4c	97.4a	95.6a
	PrBe3	86.7b	97.7b	27.3b	15.7b	12.2a	34.3a	27.5b	8.8b	29.0b	2.4b	30.8d	32.2d
	PrBe10	45.0c	52.6d	13.9c	8.4c	6.5b	23.5b	18.0c	5.8c	20.8c	1.9b	59.8d	60.7c
	PrBe3+PrBe10	21.1d	24.8e	6.0d	3.8d	2.2c	5.8c	7.0d	2.5d	4.8d	0.5c	84.5b	84.1b
	2PrBe+PeCBBz	1.2e	2.7f	0.3e	0.1e	0.8c	2.3cd	0.5e	0.1e	0.0d	0.0c	99.0a	97.7a
	2PrBe+MeCBBz	1.5e	3.8f	0.0e	0.0e	0.5c	1.5d	0.0e	0.0e	0.3d	0.1c	99.1a	98.2a
	W+	132.8a	150.9a	40.8a	28.8a	14.8a	34.9a	35.1a	14.1a	40.6a	5.6a		

CB, Cyhalofop butyl; Py, Propyrisulfuron; CBPy, Cyhalofop butyl + propyrisulfuron; PrBe3, Pretilachlor + bensulfuron applied at 3 d before transplanting; PrBe10, applied at 10 d after transplanting; PrBe3+PrBe10, two applications of pretilachor+bensulfuron (at 3 [PrBe3] and 10 [PrBe10] d]; 2PrBe+PeCBBz, two applications of pretilachor+bensulfuron (at 3 [PrBe3] and 10 [PrBe10] d) plus penosulam plus CB plus bentazone; 2PrBe+MeCBBz, two applications of pretilachor+bensulfuron (at 3 [PrBe3] and 10 [PrBe10] d) plus metamifop plus CB plus BZ; W+, nontreated weedy check. Different letters within the same column and year indicate significant differences at P = 0.05.

### 3.4 Yield and economic analysis

**Figure 5:**
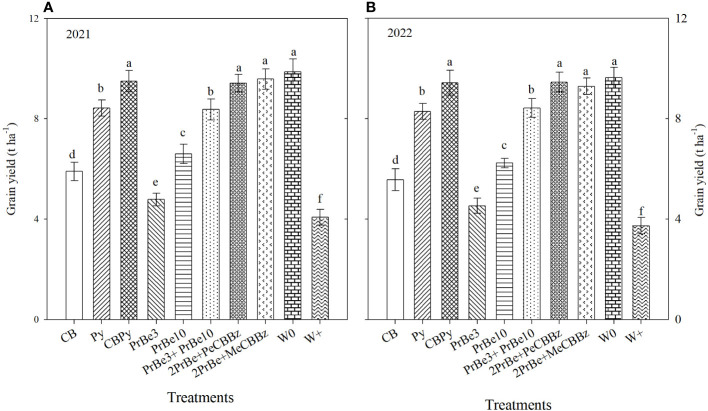
Effects of different herbicide treatments on grain yields of mechanically transplanted Nanjing 9108 rice in **(A)** 2021 and **(B)** 2022. CB, Cyhalofop butyl; Py, Propyrisulfuron; CBPy, Cyhalofop butyl + propyrisulfuron; PrBe3, Pretilachlor + bensulfuron applied at 3 d before transplanting; PrBe10, applied at 10 d after transplanting; PrBe3+PrBe10, two applications of pretilachor+bensulfuron (at 3 [PrBe3] and 10 [PrBe10] d]; 2PrBe+PeCBBz, two applications of pretilachor+bensulfuron (at 3 [PrBe3] and 10 [PrBe10] d) plus penosulam plus CB plus bentazone; 2PrBe+MeCBBz, two applications of pretilachor+bensulfuron (at 3 [PrBe3] and 10 [PrBe10] d) plus metamifop plus CB plus BZ; W0, plots kept weed-free by frequent manual weeding; W+, nontreated weedy check. Different letters indicate a significant difference at the 0.05 probability level.

The highest grain yield (9.63–9.88 t ha^−1^) was in W0, and different herbicide treatments strongly influenced grain yield. Compared with W+, herbicide treatments increased yields 17.3% to 135.2% in 2021 and 21.5% to 153.1% in 2022 (**Figure 5**).

In 2021, yields in CBPy (9.49 t ha^–1^), 2PrBe+PeCBBz (9.42 t ha^–1^), and 2PrBe+MeCBBz (9.58 t ha^–1^) were significantly higher than those in other treatments (**Figure 5**). Compared with W0, grain yields in the other herbicide treatments decreased significantly. Among those treatments, the greatest decrease in yield was in PrBe 3 (51.6%), followed by that in CB (40.3%), PrBe 10 (33.2%), PrBe3+PrBe 10 (15.2%), and Py (14.7%). Similar to 2021, in 2022, there was no difference in yield among CBPy, 2PrBe+PeCBBz, 2PrBe+MeCBBz, and W0, whereas yields in all other herbicide treatments were significantly lower than that in W0. The decrease in yield was in the order PrBe 3 > CB > PrBe 10 > Py > PrBe 3+PrBe 10 (**Figure 5**).

Production value, application of herbicide dose, costs of weeding and other practices and supplies, total cost, and economic benefit are provided in **Table 6**. Herbicide dose varied greatly among herbicide treatments, with the highest dose in 2PrBe+MeCBBz (3,090 g a.i. ha^–1^), followed by that in 2PrBe+PeCBBz (2,965 g a.i. ha^–1^), PrBe 3+PrBe 10 (1,200 g a.i. ha^–1^), PrBe 3 and PrBe 10 (600 g a.i. ha^–1^), CBPy (385.5 g a.i. ha^–1^), and CB (300 _g_ a.i. ha^–1^). The lowest dose was in Py (85.5 g a.i. ha^–1^). Cost of weeding was in an order of 2PrBe+PeCBBz > 2PrBe+MeCBBz > CBPy > Py > PrBe 3+PrBe 10 > PrBe 3 and PrBe 10 > CB. Based on rice price and other expenditures (**Table 6**), maximum mean net economic benefit was recorded in CBPy (17,932 Yuan ha^−1^), followed by that in 2PrBe+MeCBBz (17,383 Yuan ha^−1^), 2PrBe+PeCBBz (17,353 Yuan ha^−1^), PrBe 3+PrBe 10 (14,655 Yuan ha^−1^), Py (14,502 Yuan ha^−1^), PrBe 10 (8,469 Yuan ha^−1^), CB (6,321 Yuan ha^−1^), and PrBe 3 (2,805 Yuan ha^−1^), with the lowest benefit in W+ (555 Yuan ha^−1^) (**Table 6**).

**Table 6:** Average yields, production values, costs, and net economic benefits of different herbicide treatments in mechanically transplanted rice.

Treatment	Yield (t ha^–1^)	Production value (Yuan ha^−1^)	Application of herbicide dose (g a.i. ha^−1^)	Cost of weeding (Yuan ha^−1^)	Cost of seed, fertilizer, soil preparation, transplanting, pest control, and harvest (Yuan ha^−1^)	Total cost (Yuan ha^−1^)	Economic benefit (Yuan ha^−1^) ($ ha^–1^)	
CB	5.73	18336	300	90	11925	12015	6321	881.78
Py	8.36	26752	85.5	325	11925	12250	14502	2023.03
CBPy	9.32	30272	385.5	415	11925	12340	17484	2439.01
PrBe3	4.65	14880	600	150	11925	12075	2805	391.30
PrBe10	6.42	20544	600	150	11925	12075	8469	1181.43
PrBe3+PrBe10	8.4	26880	1200	300	11925	12225	14655	2044.37
2PrBe+PeCBBz	9.34	30208	2965	930	11925	12855	17033	2376.10
2PrBe+MeCBBz	9.39	30208	3090	900	11925	12825	17223	2402.61
W0	9.70	31200	0	7500	11925	19425	11615	1620.29
W+	3.90	12480	0	0	11925	11925	555	77.42

CB, Cyhalofop butyl; Py, Propyrisulfuron; CBPy, Cyhalofop butyl + propyrisulfuron; PrBe3, Pretilachlor + bensulfuron applied at 3 d before transplanting; PrBe10, applied at 10 d after transplanting; PrBe3+PrBe10, two applications of pretilachor+bensulfuron (at 3 [PrBe3] and 10 [PrBe10] d]; 2PrBe+PeCBBz, two applications of pretilachor+bensulfuron (at 3 [PrBe3] and 10 [PrBe10] d) plus penosulam plus CB plus bentazone; 2PrBe+MeCBBz, two applications of pretilachor+bensulfuron (at 3 [PrBe3] and 10 [PrBe10] d) plus metamifop plus CB plus BZ; W0, plots kept weed-free by frequent manual weeding; W+, nontreated weedy check.

## 4 Discussion

Responses of test plants to different herbicide treatments are assessed using visual phytotoxicity rating. The visual phytotoxicity rating scale developed by Rahman et al. (2011) considers chlorosis in assessing plant symptoms. According to that scale, all combinations of herbicides at the recommended rates of application (**Table 1**) resulted in a visual phytotoxicity rating of 1 (green shoots and leaves) for Nanjing 9108. However, Py application (85.5 g a.i. ha^−1^) alone or in combination reduced rice plant height by 11.3% to 17.6% at 20 and 30 DAT. These results are consistent with previous studies in which Py reduced rice plant height by 11.4% to 60.1% with application rates of 12.5–200 g a.i. ha^−1^ (Salamanez et al., 2015a; Salamanez et al., 2015b). However, in the present study, the measurement period was longer than that in previous studies, and by 28 d after Py spraying (40 DAT), there was no difference in plant height. Therefore, application of Py did not affect final rice plant height (**Figure 2**).

Propyrisulfuron is widely used to control annual or perennial weeds in paddy field, dry land, and other cultivated lands due to its high safety, high efficiency, broad-spectrum and high selectivity (Sarmah and Sabadie, 2002; Dong et al., 2022). Ikeda et al. (2011) reported that Py effectively controlled *Echinochloa* species (grasses), *Schoenoplectus juncoides*, *C. serotinus* (sedge), and *M. vaginalis* (broadleaf) at 70 and 140 g a.i. ha^−1^ with good selectivity to rice. It also has a wide insecticidal spectrum, a long efficacy period, and high biosafety for human, birds, and aquatic and other organisms (Dong et al., 2022). In the present study, high herbicide activity of Py was confirmed against species of the grass *Echinochloa*, the broadleaf weeds *M. vaginalis* and *A. multiflora*, and the sedge *C. difformis*. However, Py nearly had no herbicide activity against *L. chinensis*, indicating that Py cannot be used alone in mechanically transplanted fields. The combination CBPy showed high weed control efficiency and greatly reduced both weed number and dry weight by more than 95%. However, the level of control was slightly lower than that in 2PrBe+PeCBBz and 2PrBe+MeCBBz (above 98%), suggesting some weeds still occurred at the late stage of rice growth. Notably, those weeds that failed to seed would help deplete the weed seed bank.

In rice–wheat and rice–oilseed rape rotations in China, farmers often face the challenge of rapidly establishing rice crops due to limited labor availability (Zhang et al., 2008; Luo et al., 2019). Therefore, farmers often do not have time to apply herbicides, thereby missing the optimal time for weed control and resulting in increased later weeding efforts. Propyrisulfuron can be used not only as a pre-emergence herbicide but also as a post-emergence herbicide to control weed populations in plants with three or fewer leaves. The time from weed seedling emergence to the 3-leaf age is approximately 12–14 days, which is very useful for farmers, who can concentrate on transplanting rice seedlings. However, in early experiments, when the number of weed leaves was greater than 3, the control effect of Py decreased significantly. Therefore, for optimal weed control, the application time of Py in rice should not exceed 14 DAT.

The application of CBPy reduces the number of herbicide applications by 1-2 times, addressing labor shortages and reducing herbicide application dose. With such benefits, it is curious why the herbicide has not been widely promoted in China. Determined in a previous investigation, a higher price of Py than that of PrBe might be one reason. In addition, farmers have formed a habit of using the herbicide formula of PrBe as a pre-emergent herbicide combination, which also provides good weed control. Ultimately, farmers primarily grow rice for the net profit. In the present study, compared with W0, similar yields were obtained in CBPy, 2PrBe+PeCBBz, and 2PrBe+MeCBBzs. However, the highest net profit was in CBPy, which was significantly higher than that in 2PrBe+PeCBBz and 2PrBe+MeCBBz. Hence, given CBPy’s effective weed control and its ability to yield the highest net profit, it should be actively promoted as a preferred weed control strategy.

The results have demonstrated that CBPy offers effective weed control, labor and cost savings, and maintains high yield in mechanically transplanted rice fields. However, one potential issue with CBPy is its temporary inhibitory effect on rice height, although rice plants recover quickly from this. This temporary setback in rice height may not be acceptable to some farmers. Therefore, finding a solution to address this concern is crucial. Switching from spraying to broadcasting the herbicide may be a viable approach to prevent rice damage while still benefiting from effective weed control.

In conclusion, the field experiments showed that in mechanically transplanted rice, the herbicide treatments effectively reduced average weed density by 74.2% and average weed biomass by 74.6%. Treatments CBPy, 2PrBe+PeCBBz, and 2PrBe+MeCBBz had the highest total weed density control efficacy and total weed dry weight control efficacy. In addition, compared with W+, CBPy, 2PrBe+PeCBBz, and 2PrBe+MeCBBz also displayed relatively higher yields. Furthermore, no significant difference was observed when these treatments were compared with W0. The highest net economic gain was in CBPy, because it reduced herbicide dose and number of applications and thus weeding costs and also provided good weed control.

## Data availability statement

The original contributions presented in the study are included in the article/**Supplementary Material**. Further inquiries can be directed to the corresponding author.

## Author contributions

ZZ: Investigation, Writing – original draft, Writing – review & editing. HW: Investigation, Methodology, Conceptualization, Data curation. TG: Investigation, Methodology, Data curation, Writing – original draft. JC: Conceptualization, Data curation, Methodology, Writing – original draft. YL: Formal Analysis, Conceptualization, Investigation, Writing – original draft. GL: Formal Analysis, Software, Writing – original draft.
