# Supplementary material for: Propyrisulfuron plus cyhalofop butyl as one-shot herbicides provide high weed control efficiency and net economic performance in mechanically transplanted rice
Source: Front Plant Sci. 2023 Oct 18;14:1281931. doi: 10.3389/fpls.2023.1281931 (PMC10619164; doi:10.3389/fpls.2023.1281931)
Supplement: Supplementary file 1 [file Table_1.docx]

Supplemental Table1. Analysis-of-variance (F-values) for rice grain yield, rice height, weed density and weed biomass between years and treatments

| Source of variation | df | Rice grain Yield | Rice height | Weed density | Weed biomass |
| --- | --- | --- | --- | --- | --- |
| Year(Y) | 1 | 1.24^ns^ | 1.04^ns^ | 1.88^ns^ | 2.48^ns^ |
| Treatment (T) | 9 | 609.79^**^ | 2.01^ns^ | 1274.21^**^ | 1010.58^**^ |
| Y×T | 9 | 0.8^ns^ | 1.27^ns^ | 1.26^ns^ | 1.66^ns^ |

** represents significance at the *P* = 0.05 and *P* = 0.01 level, respectively, and ns denotes non-significance (*P* > 0.05). The experiment was in a complete randomized block design with four replicates.
